# Novel Biogenic Silver Nanoparticle-Induced Reactive Oxygen Species Inhibit the Biofilm Formation and Virulence Activities of Methicillin-Resistant *Staphylococcus aureus* (MRSA) Strain

**DOI:** 10.3389/fbioe.2020.00433

**Published:** 2020-05-25

**Authors:** Reham Samir Hamida, Mohamed Abdelaal Ali, Doaa A. Goda, Mahmoud Ibrahim Khalil, Mayasar Ibrahim Al-Zaban

**Affiliations:** ^1^Molecular Biology Unit, Department of Zoology, Faculty of Science, Alexandria University, Alexandria, Egypt; ^2^Biotechnology Unit, Department of Plant Production, College of Food and Agriculture Science, King Saud University, Riyadh, Saudi Arabia; ^3^Bioprocess Development Department, Genetic Engineering and Biotechnology Research Institute (GEBRI), City of Scientific Research and Technological Applications (SRTA-City), Alexandria, Egypt; ^4^Department of Biological Sciences, Faculty of Science, Beirut Arab University, Beirut, Lebanon; ^5^Department of Biology, College of Science, Princess Nourah bint Abdulrahman University, Riyadh, Saudi Arabia

**Keywords:** *MRSA*, inhibitory activity, *Desertifilum* sp., bacteria, silver ion, silver nanoparticles

## Abstract

Emerging antibiotic-resistant bacteria result in increased mortality and have negative economic impacts. It is necessary to discover new strategies to create alternative antibacterial agents that suppress the bacterial resistance mechanism and limit the spread of serious infectious bacterial diseases. Silver nanoparticles may represent a new medicinal agents as alternative antibiotics affect different bacterial mechanisms such as virulence and resistance. In addition to that of silver nitrate (AgNO_3_) and ampicillin, for the first time, the inhibitory effect of silver nanoparticles synthesized using *Desertifilum* sp. (D-SNPs) was evaluated against five pathogenic bacteria using the agar well diffusion method. Also, the influence of D-SNPs and AgNO_3_ on bacterial antioxidant and metabolic activities was studied. The antibacterial activity of D-SNPs and AgNO_3_ against methicillin-resistant *Staphylococcus aureus* (*MRSA*) strains was studied at the morphological and molecular level. D-SNPs and AgNO_3_ have the ability to inhibit the growth of the five bacterial strains and resulted in an imbalance in the CAT, GSH, GPx and ATPase levels. *MRSA* treated with D-SNPs and AgNO_3_ showed different morphological changes such as apoptotic bodies formation and cell wall damage. Moreover, both caused genotoxicity and denaturation of *MRSA* cellular proteins. Additionally, TEM micrographs showed the distribution of SNPs synthesized by *MRSA*. This result shows the ability of *MRSA* to reduce silver nitrate into silver nanoparticles. These data indicate that D-SNPs may be a significant alternative antibacterial agent against different bacteria, especially MDR bacteria, by targeting the virulence mechanism and biofilm formation, leading to bacterial death.

## Introduction

Many pathogenic bacteria have become a principle cause of serious infectious diseases, and with the evolution of multi-drug-resistant (MDR) bacteria, the problem has become more complicated (Brown and Wright, 2016). For example, gram-positive *Streptococcus mutans* is the most well-known bacterium causing dental caries (Metwalli et al., 2013). Among gram-negative bacteria, *Salmonella typhimurium* is one of the most common bacterial strains causing NTS infections related to human illnesses such as acute gastroenteritis (Scallan et al., 2011), while *Escherichia coli* is one of the most common bacteria responsible for a wide range of infections such as cholecystitis and urinary tract infection (Lim et al., 2010). After *E. coli*, *Klebsiella pneumonia* is the second most common disease-causing gram-negative bacterium, causing invasive infections such as pneumonia and meningitis (Podschun and Ullmann, 1998; Vading et al., 2018).

The multi-drug-resistant bacteria problem is growing rapidly due to the uncontrolled and indiscriminate usage of antibiotics for the treatment of bacterial infections (Yah and Simate, 2015). The overloading usage of antibiotics can result in issues such as MDR bacterial spread, prolonged infection treatment and high mortality risk (Yah and Simate, 2015). Both gram-negative and gram-positive bacteria include members that are distinguished as MDR bacteria or those sensitive to antibiotics (Cillóniz et al., 2019; Koulenti et al., 2019). *S. aureus* is considered a leading cause of morbidity and mortality (World Health Organization, 2017), especially with the rapid emergence of *methicillin-resistant Staphylococcus aureus* (*MRSA*) strains, which are resistant to all known β-lactam antibiotics (Boucher et al., 2009), as well as reports that clinical *MRSA* strains are also resistant to vancomycin, linezolid, and daptomycin (Nannini et al., 2010). Endocarditis, osteomyelitis, necrotizing pneumonia and sepsis infections occur when these bacteria disseminate into the blood stream (Lowy, 1998).

The appearance of the phenomenon of MDR bacteria has motivated researchers to search for solutions to explain and solve this problem as well as produce efficient alternatives to antibiotics (Gurunathan et al., 2018). These alternatives are distinguished by their potential to decrease bacterial resistance mechanisms and/or to serve as anti-virulence agents to act on pathogen virulence (Wright and Sutherland, 2007; Maura et al., 2016). One of these strategies to overcome the spread of MDR bacteria is nanotechnology, which aims to synthesize nanoparticles (NPs) featuring unique physicochemical and biological properties including anticancer (Bin-Meferij and Hamida, 2019), anti-inflammatory, and antimicrobial activity (Yallappa et al., 2016; Jagielski et al., 2018; Jalal et al., 2019). Green nanotechnology is modern science that depends on natural sources such as plants (Raut et al., 2010), bacteria (Saravanan et al., 2017), mushrooms (Debnath et al., 2019), and cyanobacteria to fabricate NPs from metals (Bin-Meferij and Hamida, 2019). The green synthesis of nanoparticles is an environmentally friendly process with low cost and energy savings (Cicci et al., 2017). Biogenic NPs are applied in different fields such as medicine, biotechnology and industry (Kubik et al., 2005). For example, gold NPs are used in photothermal therapy for cancer diseases (Ali et al., 2019); silicon nanoparticles are used in cell imaging (Guo et al., 2019); and titanium dioxide NPs are applied in photodynamic and sonodynamic therapy (Bogdan et al., 2017).

Silver nanoparticles (SNPs) are one of the most important NPs that play significant roles in biomedical applications such as wound healing, cell imaging, diagnosis, disease treatment, and contraceptive devices (Naidu Krishna et al., 2015). Furthermore, SNPs have been used in sensing, drug discovery and tissue engineering (Narducci, 2007). In addition, as SNPs are potent anticancer agents (Murugesan et al., 2019), they are considered significant antimicrobial agents with particularly high potential to effectively treat antibiotic-resistant bacteria (Leid et al., 2011; Yah and Simate, 2015). Many publications have reported that SNPs are unique antimicrobial agents against bacteria, fungi and viruses (Lu et al., 2013; Mori et al., 2013; Ouda, 2014). SNPs significantly suppress the growth of various antibiotic-resistant bacteria such as *Staphylococcus aureus* and *Pseudomonas aeruginosa* that cause mastitis infection (Leid et al., 2011).

The mechanism of action of SNPs against bacteria is still poorly understood. Yuan et al. (2017) showed that the lethal activity of SNPs against bacteria is due to the reactivity of SNPs with bacterial biocompounds such as proteins, sugars, DNA and enzymes, leading to genetic mutation and structural alterations to the bacterial cell wall and membrane as well as proteins, ultimately causing bacterial inhibition of growth and cell death.

Thus, developing and synthesizing novel biogenic SNPs as alternative antibacterial materials that enhance bacterial death is a promising strategy. In the current investigation, for the first time, we aimed to screen the inhibitory activity of biogenic SNPs, which were synthesized previously by our team by using a novel cyanobacteria strain *Desertifilum* IPPAS B-1220 (Hamida et al., 2020), against five pathogenic gram-negative and gram-positive bacteria, namely, *Escherichia coli* ATCC 25922, *Salmonella typhimurium* ATCC 14028, *Klebsiella pneumoniae* (clinical isolate), *Streptococcus mutans* and *methicillin-resistant Staphylococcus aureus* (*MRSA*) clinical isolates. Moreover, for the first time, we demonstrated the ability of *MRSA* bacteria to fabricate SNPs from silver nitrate (AgNO_3_). Finally, we added a new mechanism to explain the molecular basis of action of D-SNPs and silver ions against *MRSA*.

## Materials and Methods

### Materials

Silver nitrate (AgNO_3_), bacterial culture materials, and chemicals related to evaluating enzyme activities were purchased from Sigma-Aldrich (St. Louis, MO, United States), and PiBind resin was purchased from Expedeon, San Diego, United States.

### Preparation D-SNP Stock Solution

To prepare stock solutions of D-SNPs and silver nitrate powders, 1.54 mg of the powder was dissolved in 1 ml of distilled water.

### Antibacterial Activity

#### Bacterial Culture

Five pathogenic bacterial strains included gram-negative and gram-positive bacteria were obtained from City of Scientific Research and Technological Applications and Almery University Hospital (Alexandria, Egypt). These groups of bacteria included *Escherichia coli* ATCC 25922, *Salmonella typhimurium* ATCC 14028, drug-resistance *Klebsiella pneumoniae* (clinical isolate), *Streptococcus mutans* and *methicillin-resistant Staphylococcus aureus* (*MRSA*) (clinical isolates). Ten microliters were taken from glyceride bacteria stocks and seeded into Luria-Bertani (LB) broth for 24 h at 37°C. Fresh bacterial cultures were obtained at a concentration of 0.5 McFarland scale (10^4^ CFU/mL). Then, fresh LB agar plates were prepared, and 50 μL of each bacterial strain was poured and gently spread on the agar plates. After spreading the bacteria on the plates, four 8 mm wells were made in all plates for further experiments with the treatments.

#### Agar Well Diffusion Assay for D-SNPs and Silver Nitrate

To assess the inhibitory activity of D-SNPs and AgNO_3_ against five pathogenic bacteria, 100 μL of D-SNPs, AgNO_3_ (1.54 mg/mL), distilled water as a negative control and ampicillin antibiotic as a positive control were added to each agar plate well. The treated plates were incubated at 37°C for 24 h. At the end of the incubation time, the diameter of the inhibition zone (IZ) in mm was evaluated.

#### Minimum Inhibition Concentration

The minimum inhibition concentration (MIC) refers to the lowest concentration of antimicrobial agent at which bacterial growth is inhibited. To determine the MIC, the serial dilution method was used. In brief, different concentrations of D-SNPs and AgNO_3_ were prepared (1.8, 1.5, 1.2, 0.9, 0.6, and 0.3 mg/mL) and (2.4, 2.1, 1.8, 1.5, 1.2, 0.9, 0.6, and 0.3 mg/mL) respectively. In 96-well plates, 100 μL/well of bacterial suspension (10^4^ CFU/mL) was subjected to 100 μL of different concentrations of both D-SNPs and AgNO_3_ and then incubated for 24 h at 37°C. After 24 h, the bacterial turbidity in comparison with the 0.5 McFarland media turbidity was checked with the naked eye. To ensure the previous step, 10 μL of the resultant MIC values determined was tested against each bacterial strain using the agar well diffusion method.

#### Minimum Bactericidal Concentration

The minimum bactericidal concentration (MBC) is termed the lowest concentration of antimicrobial drugs that kill bacteria. To assess the MBC values, different concentrations of D-SNPs and AgNO_3_ (1.8, 1.5, 1.2, 0.9, 0.6, and 0.3 mg/mL) and (2.4, 2.1, 1.8, 1.5, 1.2, 0.9, 0.6, and 0.3 mg/mL) respectively were prepared, and 100 μL of each treatment concentration was added to 100 μL of each bacterial strain (10^4^ CFU/mL) and incubated for 24 h at 37°C. After 24 h, the bacterial turbidity in comparison with the 0.5 McFarland media turbidity was checked with the naked eye. To ensure the previous step, the resultant MBC values determined were tested against each bacterial strain using the agar well diffusion method.

### Estimation of Antioxidant Activity and Metabolic Toxicity

To evaluate the effect of D-SNPs and AgNO_3_ on the antioxidant activity, the bacterial cells before and after treatment with D-SNPs and silver nitrate were incubated at ambient temperature for 24 h. Then, the cells were centrifuged at 10,000 rpm for 5 min, and the pellets were washed with phosphate-buffered saline (PBS) and lysed using a sonicator (IKAT 10 basic sonicator, United States). Oxidative stress markers such as GPx, GSH, and CAT were assayed as follows:

#### Measurement of Catalase (CAT) Level

Catalase enzyme activity in the supernatants was evaluated spectrophotometrically (UV 2505 spectrophotometer, Los Angeles, United States) by measuring the drop in hydrogen peroxide (H_2_O_2_) concentration at 240 nm. In a quartz cuvette, 10 μL of supernatant was added to 2.89 mL of PBS (pH 7.4) at time 0. After adding 0.1 mL of 300 mM hydrogen peroxide to the previous mixture, the absorbance was measured at 240 nm for 5 min at 1-min intervals. The catalase activity was expressed in terms of mmol/min mg protein (Mal et al., 2013).

#### Estimation of Glutathione Peroxidase (GPx) Level

The method mentioned by Paglia and Valentine (1967) was applied to measure the activity of GPx antioxidant. In brief, the samples were added to the reaction mixture (50 mM potassium phosphate buffer (pH 7.0), 1 mM EDTA, 1 mM sodium azide, 0.2 mM NADPH, 1 U glutathione reductase, and 1 mM reduced glutathione) and allowed to equilibrate for 5 min at 25°C. The reaction was started by adding 0.1° mL of 2.5 mM H_2_O_2_. Absorbance was measured at 340 nm for 5 min. Values were expressed as *n* mol of NADPH oxidized to NADP by using the extinction coefficient of 6.2 × 10^3^ M^–1^ cm^–1^ at 340 nm. The activity of GPx was expressed in terms of *n* mol NADPH consumed/min/mg protein (Chakraborty et al., 2012).

#### Evaluation of Glutathione (GSH) Level

To evaluate the GSH level in samples with or without D-SNP and AgNO_3_ treatments, the supernatant was mixed with a solution of cold (4°C) 320 mM sulfosalicylic acid, 28 mM L-ascorbic acid, and 4 mM EDTA for protein precipitation. Then, the mixture was centrifuged at 27,000 × g (Hettich Zentrifugen, mikro 200, Germany) for 15 min at 4°C to remove the precipitated proteins, and the clear supernatants were used to evaluate the GSH concentration through a GSH assay kit based on the colorimetric method described by Beutler (1963). This reaction depends on the reduction of 5,5′-dithiobis (2-nitrobenzoic acid) (DTNB, dissolved in 25 mM PBS, pH 7.0) by the reduced glutathione to give a yellow product measured at 405 nm. The GSH content was expressed as μM GSH per milligram.

#### Measurement of ATPase Activity

The influence of D-SNPs and silver nitrate on the ATPase activity of five selected bacteria was determined using a colorimetric ATPase assay according to the method described by Andrés and Fierro (2010). Ten microliters of supernatant was incubated with 10 μL of PiBind resin to remove the free inorganic phosphate (Pi). The amount of Pi released was calculated by spectrophotometry (UV 2505 spectrophotometer, Los Angeles, United States) at A_650_. For all experiments, calibration was performed using a standard range of Pi concentrations, and data were determined for a minimum of three independent assays.

### Specimen Preparation for TEM

Bacterial specimens were fixed by immersing the cells immediately in ice-cold 4F1G [formalin-glutaraldehyde mixture (pH 7.2)] (10 mL formaldehyde 40% + 2 mL glutaraldehyde 50% + 1.16 gm sodium phosphate monobasic + 0.27 gm NaOH + distilled water up to 100 mL) and were rinsed in 0.1 M phosphate buffer for 2 h at 4°C. This step was followed by post-fixation using 1% osmium tetroxide (OsO_4_) for 2 h at 4°C. Then, the specimens were washed with 0.1 M phosphate buffer several times for 10 min. After fixation, the specimens were serially dehydrated with graded ethanol, infiltrated in propylene oxide and embedded in an Araldite Epon mixture. Ultrathin (70 nm) sections from the selected area were cut with a glass knife on an LKB Ultramicrotome and placed on 200-mesh copper grids. The ultrathin sections after double staining with 2% uranyl acetate and lead citrate were examined using a JEOL 100 CX Electron Microscope (JEOL, Tokyo, Japan) operating at 80 kV.

### Gene Expression Analysis

In this investigation, the expression levels of transcription-repair-coupling factor (mfD), α-Hemolysin (hly) and Ag43 phase-variable biofilm formation autotransporter CP4-44 prophage (flu) genes were used as molecular markers to evaluate the influence of D-SNPs and silver nitrate via quantitative RT-PCR. Total RNA was purified from samples using TRIzol Reagent (15596026, Life Technologies, United States). The yield and quality of total RNA were determined spectrophotometrically (UV 2505 spectrophotometer, Los Angeles, United States) based on the absorbance at 260 nm and the 260/280 nm ratio, respectively. Mfd, hly, and flu mRNA were evaluated using Maximas SYBR Green/Fluorescein qPCR Master Mix by Rotor-Gene Q (Qiagen, United States). One microgram of total RNA was reverse-transcribed into single-stranded complementary DNA by using a QuantiTects Reverse Transcription Kit (Qiagen, United States) with a random primer hexamer in a two-step RT-PCR reaction in which any genomic DNA (gDNA) contamination was eliminated using gDNA Wipeout buffer. Total cDNA (30 ng) was used as a template for amplification with the specific primer pair used at a final concentration of 300 nM (Table 1). Each sample was subjected to real-time PCR in duplicate, and the mean values of the duplicates were used for subsequent analysis. Glyceraldehyde-3-phosphate dehydrogenase (GAPDH) was utilized as a housekeeping gene. Rotor-Gene Q automatically collected data and analyzed the value of the threshold cycle (Ct), which was normalized to an average Ct value of the housekeeping genes (ΔCt), and the relative expression of each representative was calculated as 2–ΔCt.

**TABLE 1:** Primers used in RT-PCR.

**Gene**	**Primers**	**References**
mfD	**F:** TCAGGAAGCTGGAAGGTAATG	Ashmore et al., 2018
	**R:** GGACCATCAAGGCGGTAAT	
flu	**F:** CACAGATACGTACAGAAAGACATTCAGG	Shakerimoghaddam and Ghaemi, 2017
	**R:** GGCTGTGGGAGTTTCTGAATTG	
hly	**F:** TGAATCCTGTCGCTAATG	Tanhaeian and Ahmadi, 2018
	**R:** TATCATCCGACCTTTCACT	

### SDS–PAGE

To estimate the influence of D-SNPs and silver nitrate on the structure of cellular proteins of *MRSA*, protein electrophoresis was performed and monitored via SDS-PAGE. Total soluble proteins of *MRSA* were extracted and purified using TriFast (Peqlab, VWR company) (isolation of RNA, DNA and protein simultaneously). The OmniPAGE Mini vertical electrophoresis unit included Power Pro 5 as a power supply (Cleaver Scientific, United Kingdom) and was used to fractionate the bacterial protein content via SERVAGel^TM^ TG PRiME^TM^ 10% (SERVA, Germany). This step was followed by staining the gel with 0.1% Coomassie blue R-250 for 2 h. Then, the gel was distained with a solution (1:3:6) of glacial acetic acid: methanol: water. Finally, a gel documentation system (Geldoc-it, UVP, United Kingdom) was applied for data analysis using Totallab analysis software version 1.0.1 (Newcastle-Upon-Tyne, United Kingdom).

### Statistical Analysis

All experiments were repeated at least three times, and the data are presented as the mean ± SEM. Prism 8.3 software (GraphPad Software Inc., La Jolla, CA, United States) was used to determine the statistically significant difference between treatments and the control through one-way analysis of variance (ANOVA). The significance of the data is represented at *P* < 0.00001, *P* < 0.0001, *P* < 0.001, and *P* < 0.01. For SNP measurement, ImageJ (National Institutes of Health, Bethesda, MD, United States) was performed.

## Results and Discussion

**FIGURE 1:**
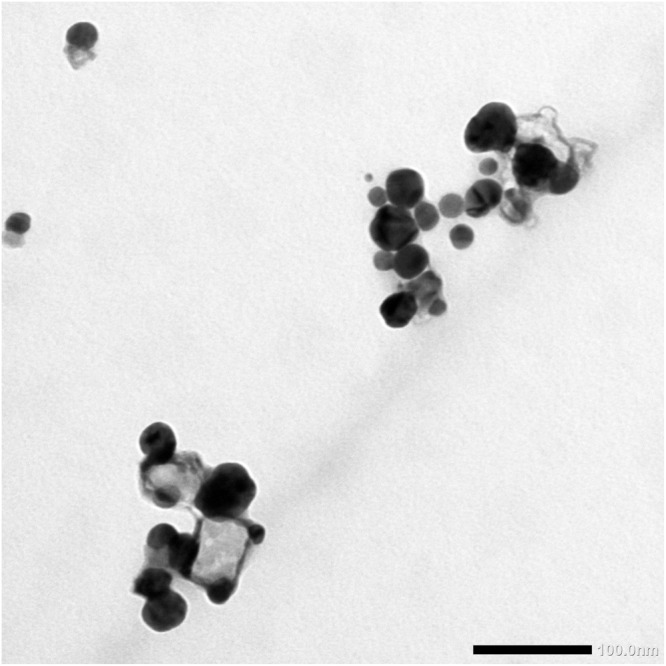
TEM micrograph of SNPs synthesized by *Desertifilum* IPPAS B-1220 demonstrating the spherical shape and distribution of D-SNPs with a size range from 4.5 to 26 nm. Scale bar, 100 nm.

Biogenic SNPs and silver ions are well known as potent antimicrobial agents against different microbes and do not exhibit any cross-resistance with antibiotics (Guggenbichler et al., 1999; Durán et al., 2016). In our recently published article (Hamida et al., 2020), we synthesized and characterized silver nanoparticles (D-SNPs) using the novel cyanobacteria species *Desertifilum* IPPAS B-1220. These nanoparticles were spherical in shape with a nano-size range from 4.5 to 26 nm (Figure 1). In the current study, in addition to AgNO_3_, D-SNPs were screened for the first time against five different gram-positive and gram-negative bacteria.

**FIGURE 2:**
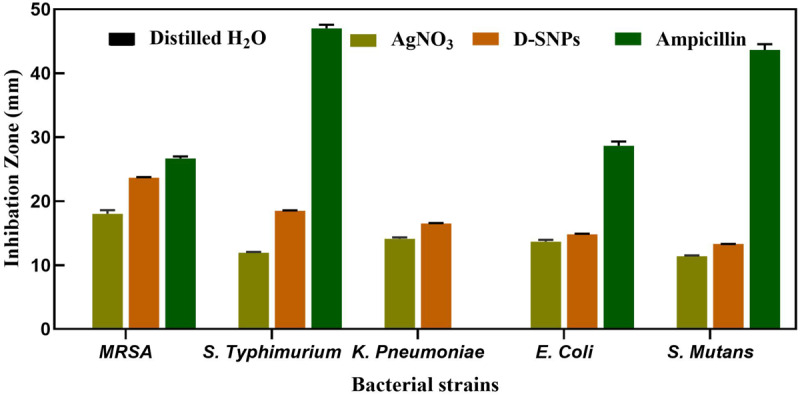
Inhibitory effect of D-SNPs, silver nitrate and ampicillin against five medically pathogenic bacteria including *Escherichia coli*, *Salmonella typhimurium*, and *Klebsiella pneumoniae*, *Streptococcus mutans*, and *Methicillin-resistant Staphylococcus aureus*.

The D-SNPs showed inhibitory effects against all selected pathogens. The inhibitory activity of D-SNPs was found to be higher (*MRSA* > *S. typhimurium* > *K. pneumoniae* > *E. coli* > *S. mutans*) than that of silver ions (Figure 2). However, D-SNPs resulted in significant toxicity against both gram-positive bacteria and gram-negative bacteria. *MRSA* was the most sensitive bacterial strain to D-SNPs and silver nitrate, with IZ diameters equal to 23.7 ± 0.08 and 18 ± 0.58, respectively; however, *MRSA* subjected to ampicillin showed an IZ diameter of 26.7 ± 0.33. *S. mutans* was the most resistant microbe toward both D-SNPs and silver nitrate, with the smallest IZ diameters of 13.3 ± 0.03 and 11.4 ± 0.12, respectively (Table 2). The convergence of IZ diameter values of D-SNPs and ampicillin antibiotic against *MRSA* indicates that D-SNPs act as a promising antibacterial agent, especially against MDR bacteria. The higher inhibitory capacity of D-SNPs against *MRSA* than that of AgNO_3_ may be because their small size and large surface area allowed more surface contact with microorganisms (Logeswari et al., 2015). Another important reason for the enhanced antibacterial activity of D-SNPs may be attributed to the *Desertifilum* biocompounds that coated SNPs (Durán et al., 2016).

**TABLE 2:** Antibacterial activity of AgNO_3_, D-SNPs, and ampicillin.

**Treatments bacteria**	**Distilled H_2__O_**	**AgNO_3_**	**D-SNPs**	**Ampicillin**
*S. Mutans*	0 ± 0	11.4 ± 0.12	13.3 ± 0.03	43.7 ± 0.88
*E. Coli*	0 ± 0	13.7 ± 0.29	14.8 ± 0.07	28.7 ± 0.67
*K. Pneumoniae*	0 ± 0	14.1 ± 0.25	16.5 ± 0.09	0 ± 0
*S. Typhimurium*	0 ± 0	11.9 ± 0.15	18.5 ± 0.06	47 ± 0.58
*MRSA*	0 ± 0	18 ± 0.58	23.7 ± 0.08	26.7 ± 0.33

Many reports have shown that SNPs are more toxic against gram-negative bacteria than against gram-positive bacteria due to the thickness of the cell wall of the latter (Slavin et al., 2017). Gram-negative bacteria are characterized by thin cell walls that allow SNPs to invade bacterial cells, while gram-positive bacteria have thick peptidoglycan cell walls that decrease the efficiency of SNPs to penetrate the cell wall (Dorobantu et al., 2015). These findings do not completely agree with the current data demonstrating the ability of D-SNPs to inhibit both gram-positive and gram-negative bacterial growth. However, our data related to *S. typhimurium*, *K. pneumoniae*, *E. coli*, and *S. mutans* agree with the results of Dorobantu et al. (2015) and Slavin et al. (2017) in which the gram-positive *S. mutans* bacterium showed the least response toward D-SNPs in comparison with that of *S. typhimurium*, *K. pneumoniae* and *E. coli*, with an IZ diameter of 13.3 ± 0.03. The lower activity of D-SNPs and AgNO_3_ toward *S. mutans* may be due to the structure of its cell wall. Based on all previous findings, the variable values of the IZ diameter after treating five bacteria with D-SNPs may not only depend on the thickness of the bacterial cell wall, as occurs in the case of *MRSA*, considering its resistance potential and the fact that *E. coli* exhibits the lowest IZ diameter value (14.8 ± 0.07) among the other tested gram-negative bacteria; the variable IZ diameter values may thus also depend on the reactivity between D-SNPs and bacteria, the mechanism of defense used by bacteria against D-SNPs, the functional groups surrounding the surface of D-SNPs and the opposite charge attraction between the surfaces of bacteria and D-SNPs. Romero-Urbina et al. (2015) showed that positively charged SNPs were more potent antibacterial agents owing to the greater affinity between these positively charged SNPs and the bacterial cell walls of *MRSA*. Salas-Orozco et al. (2019) showed that the response of bacteria toward silver nanoparticles depends on bacterial mechanisms that may be intrinsic, such as efflux pumps, downregulation of porins, and chromosomal resistance genes, or extrinsic, such as point and adaptive mutations and plasmids with resistance genes. Recent publications have reported that the enhanced inhibitory effect of biogenic silver nanoparticles may be due to neutral compounds surrounding the nanoparticles (Durán et al., 2016; Qais et al., 2019).

With the exception of *K. pneumoniae*, Table 3 shows that the MIC and MBC values of D-SNPs were lower than those of AgNO_3_. *K. pneumoniae* showed the same MIC and MBC values (1.2 and 1.5 mg/mL, respectively) for D-SNPs and AgNO_3_. These data confirm the results of the agar well diffusion method and indicate that D-SNPs were the most potent antibacterial material in comparison with AgNO_3_. Previous data have reported that plant-derived SNPs exhibited MIC values (10 mg/mL) against *E. coli*, *K. pneumoniae*, *S. typhimurium*, and *MRSA* of 2.5, 5, 2.5, and 2.5 mg/mL, respectively (Punjabi et al., 2018). These results may provide researchers with a new direction, that is, the synthesis of new alternative antibacterial drugs using cyano-nanotechnology.

**TABLE 3:** Minimum inhibition concentration (MIC) and Minimum bactericidal concentration (MBC) of silver nitrate and D-SNPs against five pathogenic bacteria.

**Bacteria**	**Treatment**					
	**AgNO_3_**			**D-SNPs**		
	**MIC (mg/mL)**	**MBC (mg/mL)**	**MIC/MBC**	**MIC (mg/mL)**	**MBC (mg/mL)**	**MIC/MBC**
*E. Coli*	1.5	2.1	0.71	1.2	1.5	0.8
*S. Typhimurium*	2.1	2.4	0.88	0.9	1.2	0.75
*S. Mutans*	1.5	1.8	0.83	0.9	1.5	0.75
*K. Pneumoniae*	1.2	1.5	0.8	1.2	1.5	0.8
*MRSA*	1.5	1.8	0.83	1.2	1.5	0.8

Many publications have tried to explain the mechanism of action of SNPs against bacteria, and some of these reports attributed the lethal effect of SNPs to their ability to induce the formation of reactive oxygen species (ROS) and metabolic toxicity (Yuan et al., 2017; Gurunathan et al., 2018). The current study screened the relationship between D-SNPs and AgNO_3_ and the levels of antioxidants such as GPx, GSH and catalase as well as the activity of ATPase.

**FIGURE 3:**
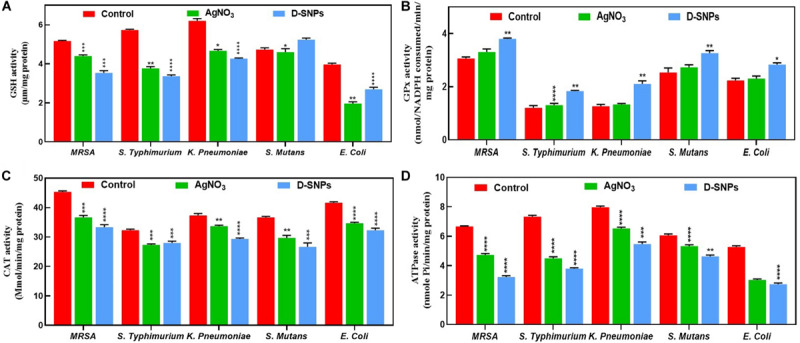
Effect of D-SNPs and silver nitrate on antioxidants and enzymes activities. **(A)** GSH. **(B)** GPx. **(C)** CAT. **(D)** ATPase. Data from at least three independent experiments performed at least in triplicate are presented as the mean SEM; P-values were calculated versus untreated bacterial cells: ****P < 0.00001, ***P < 0.0001, **P < 0.001, and *P < 0.01.

Glutathione (GSH) is an antioxidant that exists in all living organisms such as plants, animals, bacteria, fungi and archaea. GSH has the ability to protect cells from damage caused by ROS such as free radicals, peroxides, and lipid peroxides as well as those from heavy metals (Pompella et al., 2003). With the exception of that in *S. mutans*, the GSH activity in all tested bacteria was decreased after D-SNP treatment in comparison with the control and silver nitrate treatments (Figure 3A). A similar result was observed for AgNO_3_; the GSH level was decreased in the five selected bacteria, however, the effect of D-SNPs on the GSH level was stronger than that of silver nitrate. The decrease in GSH levels may be due to the D-SNPs causing intense oxidative stress by inducing ROS formation inside bacteria. This finding is consistent with data reported by Gurunathan et al. (2018), who showed that after treatment of pathogenic bacteria with silver nanoparticles, the level of antioxidant GSH was sharply decreased. Banerjee et al. (2010) reported that treatment of *Pseudomonas putida* with SNPs led to increased peroxidation with a simultaneous weakening of the antioxidant defense system.

With respect to untreated bacteria, *S. mutans* showed increased GSH levels after treatment with D-SNPs. This result indicated that *S. mutans* may be more resistant to D-SNPs by increasing the GSH antioxidant level. This finding is consistent with data obtained from the agar well diffusion assay, in which *S. mutans* was the pathogen least affected by D-SNPs. Barros et al. (2019) mentioned that some bacteria detoxify heavy metals and nanoparticles by increasing the antioxidant levels. *E. coli* showed a greater decrease in the GSH levels after treatment with silver nitrate than that after treatment with D-SNPs. This result may indicate that oxidative stress caused by silver nitrate may be more than that caused by D-SNPs. Additionally, the defense mechanism of bacteria toward heavy metals and nanoparticles depends on many factors such as the type of bacteria, their neutral habitats, the stressors in their habitat and their capacity to react to external stress (Japenga et al., 1990; Fabrega et al., 2011).

Glutathione peroxidase (GPx) is a member of the enzyme family characterized by peroxidase activity. These enzymes protect the cells from oxidative stress through the reduction of lipid hydroperoxides to their corresponding alcohols and the reduction of free hydrogen peroxide to water (Muthukumar and Nachiappan, 2010; Muthukumar et al., 2011). The GPx enzyme activity of the five bacterial strains after administration of D-SNPs and AgNO_3_ was increased in comparison with that of the control (Figure 3B); however, in comparison with AgNO_3_, the D-SNPs led to a greater increase in the GPx enzyme activity. The increase in the GPx level may be due to D-SNPs increasing the production of ROS, and the bacteria fight this stress by scavenging hydroperoxides through increased expression of the GPx enzyme. These data are in accordance with that of Barros et al., who demonstrated that exposure of *Pseudomonas* sp. M1 to both silver ions and SNPs resulted in increased GPx antioxidant levels (Barros et al., 2019). The current data corroborate data from GSH level analysis, in which the higher activity of the GPx enzyme is related to the low GSH level as GPx converts the GSH substrate into GSSG (oxidized form), affecting the GSH stores required for cellular protection against oxidative stress and inducing bacterial cell death (Yuan et al., 2017).

Catalase is a common enzyme that exists in approximately all living organisms exposed to oxygen; it catalyzes the decomposition reaction of hydrogen peroxide to water and oxygen. This enzyme plays an important role in protecting cells from oxidative stress resulting from ROS (Chelikani et al., 2004). The five bacterial strains after 24 h of treatment with D-SNPs and AgNO_3_ showed a reduction in catalase enzyme activity (Figure 3C). Barros et al. showed that the catalase enzyme activity in *P. aeruginosa* cells after a short exposure (1 h) to SNPs was increased, while after a long exposure (12–24 h), the activity of catalase was decreased (Barros et al., 2019). This finding may explain why after a short exposure to silver ions and D-SNPs, the catalase activity increases as a response to external stress in the experiment to mitigate the external danger, while with increasing exposure time, the bacteria lose their ability to detoxify the silver nanoparticles and silver ions, so the catalase activity drops. Additionally, Gurunathan et al. (2018) reported that after being treated with SNPs, *Prevotella melaninogenica* and *Arcanobacterium pyogenes* showed low catalase activity after 12 h.

In all tested bacteria with the exception of *S. typhimurium*, D-SNPs were the agent most responsible for reducing the catalase activity compared to that of AgNO_3_ and untreated bacteria. *S. typhimurium* showed a greater decrease in catalase levels after treatment with silver nitrate than that with D-SNPs. These results showed that D-SNPs may be responsible for a significant drop in antioxidant levels as a result of causing intense oxidative damage in the bacterial cell by increasing the production of ROS (Banerjee et al., 2010; Gurunathan et al., 2018). Additionally, these findings are consistent with data reported by Yuan et al. (2017) showing that biogenic SNPs caused a decrease in the catalase level of drug-resistant strains of *Staphylococcus aureus* and *Pseudomonas aeruginosa*. The observed positive association between GPx and catalase activities under D-SNP exposure suggested the contribution of these enzymes to the scavenging of peroxides/hydroperoxides. The more pronounced activities of antioxidant enzymes under D-SNP exposure were consistent with the higher toxicity of the nano-form (Barros et al., 2019). Our results showed the involvement of different antioxidants such as GPx, GSH (part of the ascorbate–glutathione cycle) and catalase in coping with the oxidative stress induced by both silver nitrate and D-SNPs. However, the greatest imbalance in antioxidant activities was related to exposure to D-SNPs, which may be due to their small size, charge and coating with *Desertifilum* biocompounds. These factors may increase the potential of D-SNPs to penetrate bacterial cells and interfere with bacterial biomolecules.

ATPase is an enzyme responsible for the decomposition of adenosine triphosphate (ATP) into adenosine diphosphate (ADP) and the release of free phosphate ions or the inverse reaction. The dephosphorylation reaction results in liberating energy, which the enzyme harnesses to drive other biochemical reactions such as anabolic processes (Riley and Peters, 1981). The five bacterial strains treated with D-SNPs and silver nitrate exhibited decreased ATPase activity compared with that of the control (Figure 3D). However, D-SNP caused a greater decrease in ATPase activity than silver nitrate did. The decrease in ATPase activity may be because D-SNPs interfere with the ATPase enzyme, causing dysfunction of this enzyme. This finding is in agreement with the results of Cui et al., who stated that gold NPs inhibit ATPase activity, leading to a decreased ATP content in *E. coli* (Cui et al., 2012).

Based on the results from the analysis of enzyme activities, the mechanism by which D-SNPs and silver ions kill bacteria may be explained by an increase in ROS formation, leading to increased oxidative stress and metabolic toxicity. This toxicity leads to damage to biomolecules such as enzymes, proteins and DNA, resulting in bacterial cell death (Gurunathan et al., 2018).

**FIGURE 4:**
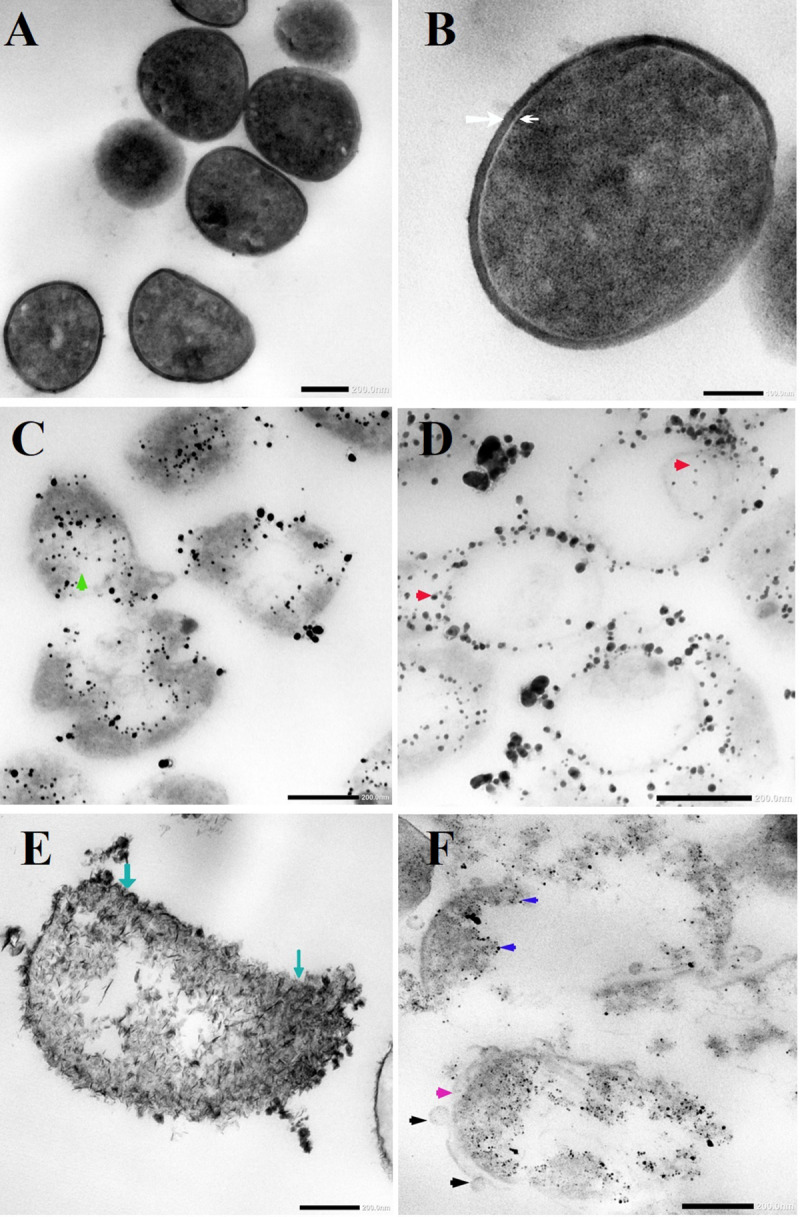
**(A,B)** TEM micrographs of untreated *MRSA* showing intact closely packed cells with thicken peptidoglycan layers cell walls (white arrow) and bacterial membrane (head arrow). **(C)** *MRSA* treated with silver nitrate appeared to undergo lysis (green arrow). **(D)** Distribution of SNPs synthesized by *MRSA* (red arrow). **(E)** *MRSA* treated with D-SNPs showing cell wall disruption with folds formation (blue arrow). **(F)** *MRSA* subjected with D-SNPs showing separation plasma membrane from the cell wall (pink arrow), secretory vacuoles (black arrow), D-SNPs inside cells (dark blue arrow) causing cytoplasmic lashing. Scale bar, 200 nm.

**FIGURE 5:**
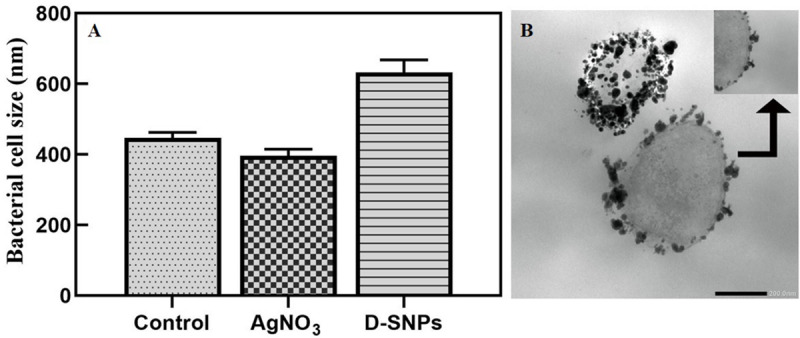
**(A)** *MRSA* cell size after treating with silver nitrate and D-SNPs. **(B)** TEM image showing the D-SNPs attached on *MRSA* cell wall. Scale bar, 200 nm.

**FIGURE 6:**
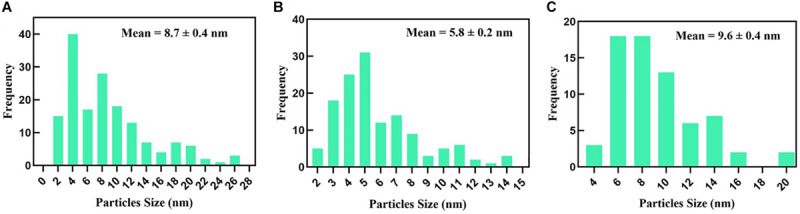
**(A)** D-SNPs size distribution outside *MRSA* cell. **(B)** D-SNPs size distribution inside *MRSA* cell. **(C)** Size distribution of SNPs synthesized by *MRSA*.

TEM micrographs showed that untreated *MRSA* cells were generally intact and closely packed, with typical cytoplasmic membranes, and their cell walls were rigid, with thickened peptidoglycan (PG) layers (Figures 4A,B). In contrast, *MRSA* cells treated with silver nitrate lost their coccal appearance and appeared to undergo lysis, leading to liberation of the cellular contents into the surrounding environment and ultimately resulting in cell disruption (Figures 4C,D). Moreover, the morphological changes after treating *MRSA* with silver nitrate included disintegration of the bacterial cells, a decrease in the electron density of the cytoplasm and in their sizes (Figure 5A). D-SNPs resulted in deformation of the *MRSA* shape, reduction in bacterial density, cell bursting, scattering of cell debris and vacuoles (Figures 4E,F). Additionally, the D-SNPs led to an increase in cell size (Figure 5A), disruption of the cell wall, damage to PG layers and induced the formation of many pores, holes and folds and cytoplasmic filaments; all these signs confirm that D-SNPs induce bacterial cell death (Figure 4F). Figure 5B demonstrates the affinity between D-SNPs and bacterial cell walls, in which clusters of D-SNPs bind to the cell walls, resulting in cytoplasmic leakage and discharge of cytoplasmic components that aid in bacterial cell lysis and induce apoptotic body formation. These findings are in agreement with those of Romero-Urbina et al. (2015), who studied the effect of positively charged SNPs on the morphology of *MRSA* and showed that SNPs induce peptidoglycan layer damage, porosity of the cell membrane and discharge of cytoplasmic material. The D-SNP and silver nitrate treatments resulted in morphological changes of *MRSA*; however, the acute changes were caused by D-SNPs. It was found that D-SNPs inside bacterial cells have a smaller size, with a size range from 2 to 14.6 nm, than that of D-SNPs outside bacterial cells, with a size range equal to 2.5–26.8 nm (Figures 6A,B). The larger size of D-SNPs outside the bacterial cells than inside the cells may be due to the agglomeration of D-SNPs outside the bacteria and indicated that the larger particles may be trapped outside while small D-SNPs easily enter the bacteria (Romero-Urbina et al., 2015). On the other hand, the TEM micrographs also showed the existence of electron dense spherical particles that may be SNPs resulting from the reduction of silver nitrate by *MRSA* (Figures 4C,D). The silver nanoparticles synthesized by *MRSA* (M-SNPs) were distributed at the plasma membrane, cytoplasm and genetic material and have sizes ranging from 3.6 to 20.6 nm, and their average size is 9.6 ± 0.4 nm (Figure 6C). It is interesting to note that this investigation represents the first report showing the ability of *MRSA* to fabricate small SNPs from silver nitrate. Jung et al. mentioned in their publication that it is common to observe the presence of electron-dense particles or precipitates around damaged bacterial cells of *S. aureus* treated with silver ions (Jung et al., 2008).

Our consideration of this result is that *MRSA* may be attempting to overcome the external stress of a heavy metal (silver nitrate); the bacteria convert the bulk material into small nanoparticles (NPs), but these NPs kill the bacteria. Wakshlak et al. (2015) called this phenomenon a “zombie effect,” in which bacteria synthesize NPs that kill them.

It was found that not all bacteria are able to perform this action, suggesting that the ability of *MRSA* to fabricate the smaller SNPs from silver nitrate may be a resistance feature that aids the bacterium. Pandian et al. (2010) reported that there are two possible mechanisms for killing bacteria with silver nitrate. First, at lower concentrations of silver nitrate, silver ions are converted to silver nanoparticles by bacterial cells, and these synthesized NPs kill the bacteria from within. However, at higher concentrations, silver nitrate induces apoptosis by increasing the hydrogen peroxide levels through inactivation of thiol group-containing proteins such as NADH dehydrogenase II or by direct binding with DNA (Pandian et al., 2010).

**FIGURE 7:**
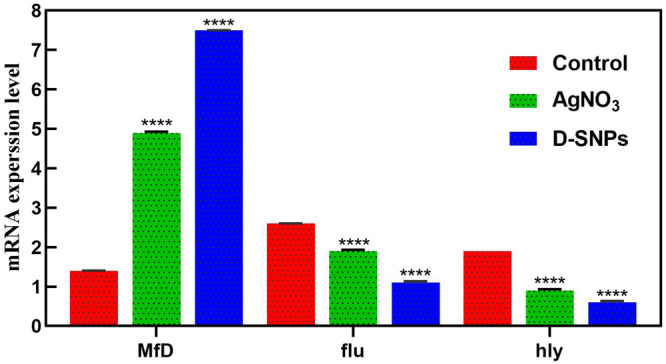
mRNA expression level of mfD, flu, hly genes of *MRSA* before and after treatment with AgNO_3_ or D-SNPs. Data from at least three independent experiments performed at least in triplicate are presented as the mean ± SEM; *P*-values were calculated versus untreated bacterial cells: ****P < 0.00001.

The change in gene expression of *MRSA* mfD, hly and flu genes was evaluated using qRT-PCR before and after treatment of *MRSA* with D-SNPs and silver nitrate at a concentration of 1.54 mg/mL. Total RNA was extracted, cDNA was created, and q real-time PCR was performed using specific primers of mfD, hly, and flu, with GAPDH as a housekeeping gene control. The data showed that the exposure of *MRSA* to D-SNPs and silver nitrate caused upregulation of mfD gene expression but downregulation of the expression of hly and flu genes (Figure 7). Moreover, the higher increase in the mRNA levels of the mfD gene may be a bacterial response to genotoxic damage caused by D-SNPs as a result of intense oxidative stress. This finding is consistent with that of Ashmore et al. (2018), who treated *E. coli* with polymer-coated SNPs and reported that upregulation of the mfD gene was a response of the bacteria to damage caused by the NPs. On the other hand, the decrease in the mRNA level of the flu gene may be due to the stress induced by D-SNPs causing loss or disruption of bacterial function such as biofilm formation, as represented here by the flu gene. The inhibitory effect of zinc NPs on the flu gene of *E. coli* was reported by Shakerimoghaddam et al. (2017). The authors showed that zinc NPs reduced the expression of the flu and fimH genes, resulting in a reduction in biofilm formation (Shakerimoghaddam and Ghaemi, 2017; Shakerimoghaddam et al., 2017).

Additionally, the downregulation of the alpha-hemolysin gene as a crucial virulence factor of the pathogen may indicate that D-SNPs affect bacterial defense mechanisms by reducing virulence and invasiveness (Soleimani and Habibi-Pirkoohi, 2017). This result showed that D-SNPs may be a promising anti-virulence agent. Das et al. (2016) showed that multi-drug-resistant *Pseudomonas aeruginosa* strains treated with SNPs exhibited downregulation of the hly gene. A recent publication reported that biogenic SNPs synthesized using *Shewanella oneidensis* act as potent anti-virulence agents by downregulating the hly gene of *E. coli* (Tanhaeian and Ahmadi, 2018).

Similarly, the upregulation of the mfD gene and downregulation of flu and hly genes of *MRSA* after exposure to silver nitrate and/or M-SNPs may be due to silver nitrate and/or M-SNPs causing genotoxicity by inducing ROS formation (Pandian et al., 2010; Barros et al., 2019). It was found that the effect of D-SNPs on the gene expression of mfD, hly and flu of *MRSA* was greater than that of silver nitrate and/or M-SNPs. This finding may be due to the features of the D-SNPs including the *Desertifilum-*derived materials that coated the particles, the particle size and the charge of the D-SNPs. These factors may facilitate the penetration through the *MRSA* plasma membrane, and attraction between D-SNPs and genetic material allowed these NPs to exhibit the highest genotoxicity as a result of intense oxidative stress (Romero-Urbina et al., 2015). Based on previous data, we supposed that D-SNPs and silver nitrate and/or M-SNPs caused genotoxicity and imbalance in the gene expression of *MRSA* through induction of oxidative stress.

**FIGURE 8:**
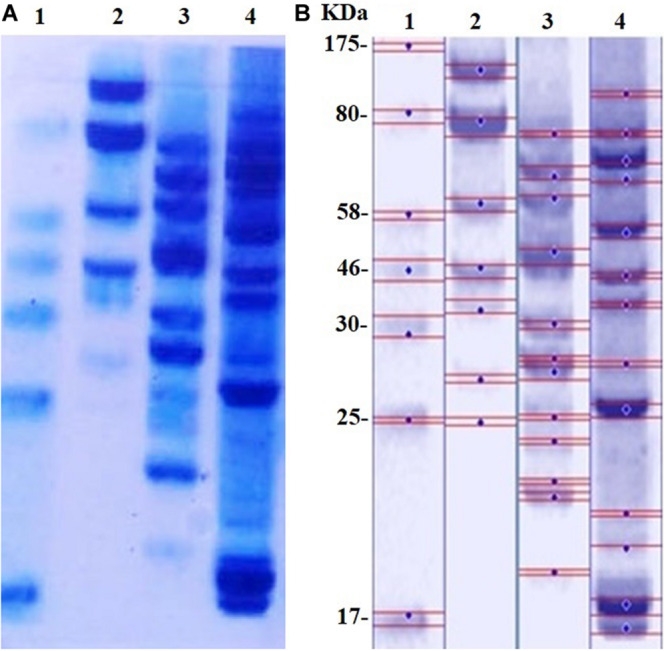
**(A)** SDS-PAGE of MRSA cells (10^4^ CFU/mL) incubated with D-SNPs or silver nitrate for 24 h. **(B)** Computerized analysis of band intensities. (1) Marker, (2) untreated *MRSA*, (3) *MRSA* treated with AgNO_3_, (4) *MRSA* treated with D-SNPs.

The SDS-PAGE results of proteins extracted from *MRSA* before and after treatment with D-SNPs and silver nitrate are shown in Figures 8A,B. Considering that of the control, the results showed that the band intensity of proteins of *MRSA* treated with D-SNPs was very thin, and new protein bands appeared. Additionally, in comparison with those of untreated bacteria, the large molecular weight protein bands from the treated bacteria were largely prevented from entering the electrophoresis gel. This result reflects that ROS formed after treating *MRSA* with D-SNPs may induce significant covalent modifications to the proteins that either caused the formation of insoluble aggregates that were too large to enter the electrophoresis gel or degradation of proteins into fragments that were too small to be detected by the technique (Wang et al., 2013). Moreover, these results may be attributed to the D-SNPs inducing the expression of new proteins as a stress response and/or preventing the synthesis of other proteins by inhibiting many translation factors. These data are in accordance with the results of Soliman et al. (2018) who reported that biogenic SNPs lead to the presence of new protein bands and increasing in band intensity of samples treated with SNPs were more than in untreated samples at the two separation speed of gel electrophoresis (4000, 14000 rpm). They mentioned that SNPs induced bacterial and fungal cells (*Bacillus* sp. and *Candida* sp. respectively) to produce new proteins as a response to this stress. Moreover, in case *E. coli* spiked with SNPs, the protein profile remain unchanged in comparing with untreated cells, however, band intensities were comparatively less than those of the control (Soliman et al., 2018). This finding implies that the affinity of D-SNPs to the thiol groups of proteins causing unfolding of the protein chain might cause cellular protein modification and degradation of the proteins (Gogoi et al., 2006; Velusamy et al., 2016). Tamiyakul et al. (2019) showed that SDS-PAGE of treated *S. aureus* and *E. coli* with SNPs coated with poly (4-styrenesulfonic acid-co-maleic acid) polymer exhibited more protein expression in comparison with untreated bacteria. They suggested that protein pattern modification results in bacterial death through interacting with metabolic function and synthesis of cell wall, nucleic acid, and protein (Tamiyakul et al., 2019). Tiwari et al. (2017) studied the effect of chemically synthesized polyvinylpyrrolidone-SNPs on sRS-307 bacterial proteins using SDS-PAGE. They showed that SNPs resulted in overexpression of 43 kDa protein while a protein with 35 kDa undergoes downregulation. Similar results were observed for silver nitrate and/or M-SNPs, but the protein bands with large electrophoretic molecular weights were slightly heavier than those from the D-SNP treatment. Additionally, the number of protein bands after treatment with silver nitrate was 12, while that in the case of D-SNP treatment was 14 (Table 4), indicating that the D-SNPs caused more fragmentation and denaturation of proteins.

**TABLE 4:** Number and intensity of protein bands of *MRSA* cells before and after treatment with D-SNPs or AgNO_3_.

**Control**			**AgNO_3_**			**D-SNPs**		
**Band No.**	**Lane%**	**MW (kd)**	**Band No.**	**Lane%**	**MW (kd)**	**Band No.**	**Lane%**	**MW (kd)**
1	1.07	135.262	1	0.63	65.839	1	0.74	100.471
2	1.37	73.579	2	1.36	58.039	2	0.48	65.839
3	1.49	58.039	3	6.53	58.039	3	2.52	58.817
4	1.53	46.769	4	1.50	51.213	4	1.91	58.039
5	1.89	35.000	5	0.98	31.991	5	1.04	55.500
6	1.21	25.761	6	0.59	27.037	6	0.50	44.459
7	1.31	24.962	7	1.64	26.124	7	0.55	36.333
			8	0.82	25.035	8	0.87	26.620
			9	0.84	24.616	9	0.84	25.132
			10	0.84	23.451	10	1.03	22.178
			11	1.05	22.847	11	1.02	20.547
			12	0.94	19.311	12	6.05	19.028
						13	2.45	16.562
						14	5.16	16.452

**FIGURE 9:**
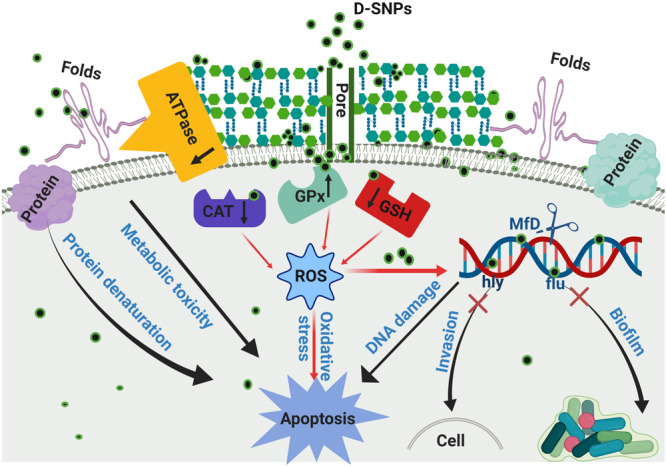
Scheme of the mechanism of action of D-SNPs against *MRSA* bacteria.

The higher toxicity of D-SNPs to *MRSA* than that of silver nitrate and/or M-SNPs may be due to the existence of *Desertifilum* biomolecules that surround the SNPs, preventing the agglomeration of SNPs, improving the ability of NPs to contact bacterial cells and promoting their antimicrobial properties (Chen et al., 2016). It has been suggested that D-SNPs cause inactivation of cellular proteins and loss of DNA replication ability by inducing ROS formation, leading to intense oxidative stress and bacterial cell death (Figure 9).

## Conclusion

Biogenic silver nanoparticles may represent a new chance in the medical fields because they can act as alternative antibiotics that affect different bacterial mechanisms such as virulence and resistance. We explored the potential of novel D-SNPs and AgNO_3_ to inhibit the growth of different pathogenic bacteria including multi-drug-resistant bacteria such as *MRSA*. It was possible to conclude that D-SNPs were the most effective compound, showing potent antibacterial, anti-biofilm formation and anti-virulence activities against bacteria. Additionally, this investigation examined for the first time the ability of *MRSA* to biofabricate smaller SNPs from silver nitrate. Furthermore, the insights of this study into how novel D-SNPs impact bacterial metabolism could provide key information on the mechanisms of action, resulting in the discovery of new therapeutic methodologies for the development of better antibacterial agents. We suggest for the first time that the mechanism of action of D-SNPs and AgNO_3_ against *MRSA* is mainly related to oxidative stress induced by the generation of ROS, which results in an imbalance of antioxidant activity leading to the oxidation of biomolecules such as DNA, enzymes, RNA and proteins as well as bacterial death. This work demonstrates that green D-SNPs are efficient antibacterial agents that may serve as alternative drugs to antibiotics, opening the door to create synergetic nano-antibiotics to address multi-drug-resistant bacteria. Other findings are needed to complete the study of D-SNPs against different bacteria and fungi and the targeting of other genes to elucidate the specific molecular mechanism of these nanoparticles against bacteria.

## Data Availability Statement

The data supporting this article are available in Figures 1–9 and Tables 1–4. The data sets analyzed in the present study are available from the corresponding author upon reasonable request.

## Author Contributions

RH, MA, DG, MK, and MA-Z contributed conception and design of the study. RH, DG, and MA-Z downloaded and organized datasets. RH performed the statistical and result analysis and wrote the first draft of the manuscript. All authors contributed to manuscript revision, read, and approved the submitted version.

## Conflict of Interest

This research was included in a request for patent 120410334 from King Abdulaziz City for Science and Technology, Saudi Arabia, submitted on 07/01/2020, titled “Antibacterial activity of biosynthesized silver nanoparticles” https://epatentsso.saip.gov.sa/.

The authors declare that the research was conducted in the absence of any commercial or financial relationships that could be construed as a potential conflict of interest.

## Abbreviations

μ g: microgram
μ L: microliter
AgNO_3_: silver nitrate
D-SNPs: silver nanoparticles synthesized by *Desertifilum* sp.
h: hour
IZ: inhibition zone
kb: kilobase
kV: kilovolt
mg: milligram
min: minute
mL: milliliter
mM: millimolar
nm: nanometer
NPs: nanoparticles
PCR: polymerase chain reaction
rpm: revolutions per minute
SNPs: silver nanoparticles
TEM: transmission electron microscope
UV: Ultraviolet-visible spectroscopy.
